# A comprehensive meta-analysis of transcriptome data to identify signature genes associated with pancreatic ductal adenocarcinoma

**DOI:** 10.1371/journal.pone.0289561

**Published:** 2024-02-07

**Authors:** Shirin Omidvar Kordshouli, Ahmad Tahmasebi, Ali Moghadam, Amin Ramezani, Ali Niazi

**Affiliations:** 1 Institute of Biotechnology, Shiraz University, Shiraz, Iran; 2 Department of Medical Biotechnology, School of Advanced Medical Sciences and Technologies, Shiraz University of Medical Sciences, Shiraz, Iran; 3 Shiraz Institute for Cancer Research, School of Medicine, Shiraz University of Medical Sciences, Shiraz, Iran; University of Nebraska Medical Center, UNITED STATES

## Abstract

**Purpose:**

Pancreatic ductal adenocarcinoma (PDAC) has a five-year survival rate of less than 5%. Absence of symptoms at primary tumor stages, as well as high aggressiveness of the tumor can lead to high mortality in cancer patients. Most patients are recognized at the advanced or metastatic stage without surgical symptom, because of the lack of reliable early diagnostic biomarkers. The objective of this work was to identify potential cancer biomarkers by integrating transcriptome data.

**Methods:**

Several transcriptomic datasets comprising of 11 microarrays were retrieved from the GEO database. After pre-processing, a meta-analysis was applied to identify differentially expressed genes (DEGs) between tumor and nontumor samples for datasets. Next, co-expression analysis, functional enrichment and survival analyses were used to determine the functional properties of DEGs and identify potential prognostic biomarkers. In addition, some regulatory factors involved in PDAC including transcription factors (TFs), protein kinases (PKs), and miRNAs were identified.

**Results:**

After applying meta-analysis, 1074 DEGs including 539 down- and 535 up-regulated genes were identified. Pathway enrichment analyzes using Gene Ontology (GO) and the Kyoto Encyclopedia of Genes and Genomes (KEGG) revealed that DEGs were significantly enriched in the HIF-1 signaling pathway and focal adhesion. The results also showed that some of the DEGs were assigned to TFs that belonged to 23 conserved families. Sixty-four PKs were identified among the DEGs that showed the CAMK family was the most abundant group. Moreover, investigation of corresponding upstream regions of DEGs identified 11 conserved sequence motifs. Furthermore, weighted gene co-expression network analysis (WGCNA) identified 8 modules, more of them were significantly enriched in Ras signaling, p53 signaling, MAPK signaling pathways. In addition, several hubs in modules were identified, including *EMP1*, *EVL*, *ELP5*, *DEF8*, *MTERF4*, *GLUP1*, *CAPN1*, *IGF1R*, *HSD17B14*, *TOM1L2* and *RAB11FIP3*. According to survival analysis, it was identified that the expression levels of two genes, *EMP1* and *RAB11FIP3* are related to prognosis.

**Conclusion:**

We identified several genes critical for PDAC based on meta-analysis and system biology approach. These genes may serve as potential targets for the treatment and prognosis of PDAC.

## Introduction

Pancreatic cancer (PC) is one of the most lethal types of cancer, with exocrine cells accounting for approximately 95 percent of cases. This type of PC is commonly known as PDAC [1], and is one of the most common malignant tumors of the gastrointestinal tract, as well as the seventh leading cause of cancer death worldwide [2], with a five-year survival rate of less than 5%. According to projections, PDAC will overtake breast and colorectal cancer as the second leading cause of cancer death by 2030. It has been observed that there is high mortality and very poor prognosis of PDAC as a result of unclear early symptoms and lack of specific molecular markers for early diagnosis. Most patients with advanced cancer are diagnosed with local invasion or distant metastasis [3]. Therefore, identification of the key genes and pathways is necessary to deepen our understanding of the molecular mechanisms of PDAC that can provide reliable biological markers and treatment targets [4].

Gene expression has also become a powerful tool in recent years for predicting the role and activity of genes. Advances in high-throughput measurement technologies and a large amount of gene expression data in public databases provide an opportunity to obtain more reliable and transparent results. Besides, using meta-analysis techniques has been increasingly employed to integrate data from different resources and is especially useful for combining several datasets related to the same disease when they are limited in size [4] for increasing statistical power [5]. In addition, exploration of interactions among genes can help to better explain the complex mechanisms of biological processes. Expression analysis identifies those genes that have similar expression patterns. Genes that express a high degree of expression are likely to be involved in a common biological process or metabolic pathway [6]. WGCNA identifies correlation patterns among the genes, detecting highly correlated gene modules and summarizing many of the hub genes and biomarkers [7]. Currently, WGCNA has been used for several types of cancer that have been associated with promising results [8].

In this study, we applied large-scale microarray data for meta-analysis to find DEGs associated with PDAC. Following that, WGCNA was used to identify the co-expression of genes. Various bioinformatic methods were also applied to help in the identification of the most important candidate genes that can be considered as potential biomarkers and therapeutic targets in PDAC.

## Methods

### Data collection

**Fig 1:**
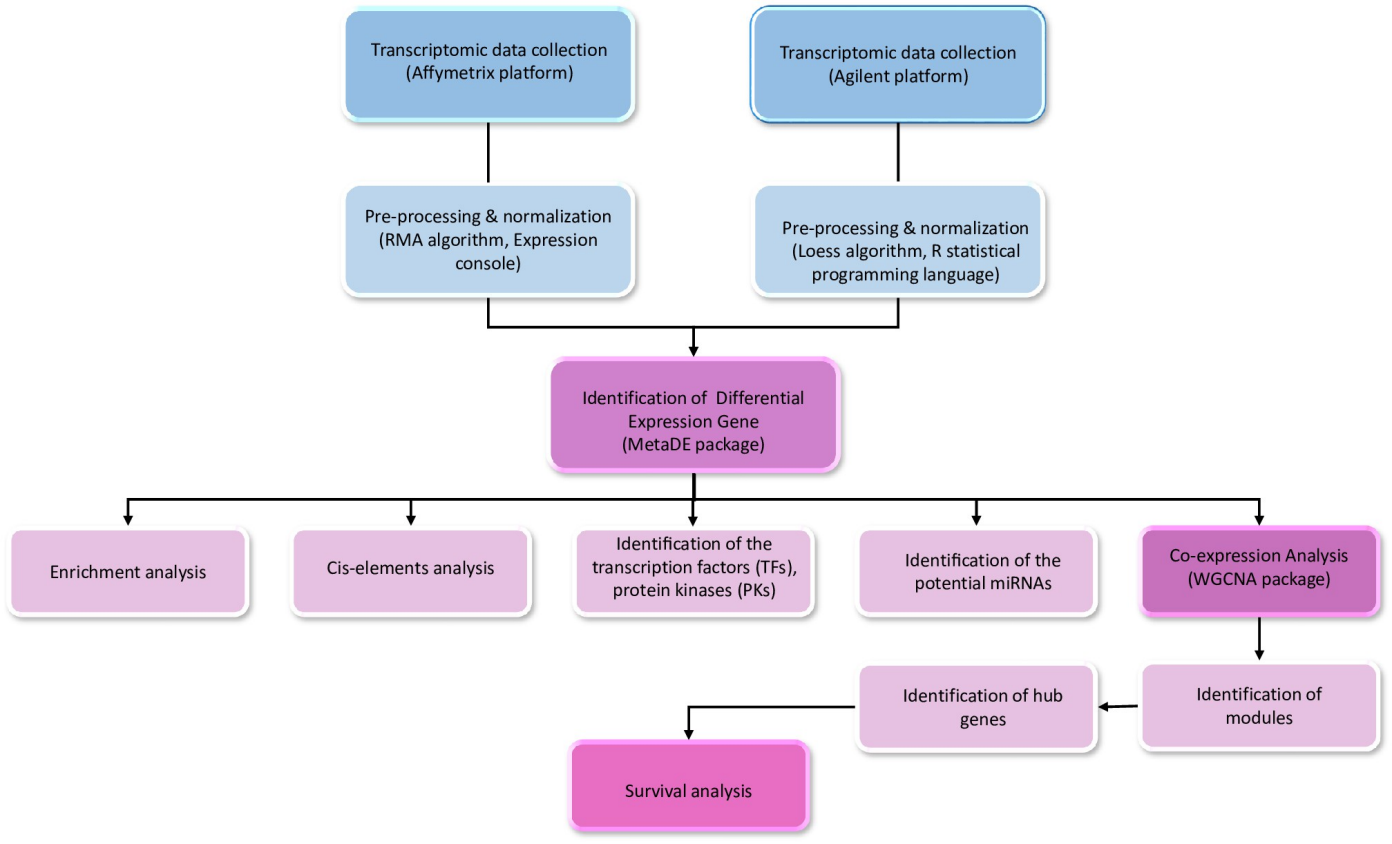
Workflow including meta-analysis and bioinformatics pipeline. Gene expression datasets of PDAC and pancreatic cancer were obtained from the GEO. The datasets were normalized and processed to identify differentially expressed genes (DEGs) between normal and tumor tissues. The significantly enriched pathways and Gene Ontology were identified through enrichment analyses. Conserved motifs and consensus cis-regulatory elements (CREs) of DEGs were detected. The WGCNA was used to cluster genes with the highest connection and identification of co-expression modules.

Microarray-based expression datasets were retrieved from the Gene Expression Omnibus (GEO) database (https://www.ncbi.nlm.nih.gov/) (Fig 1). To investigate transcriptome responses in pancreatic cancer, we used 11 datasets. We selected only datasets with both tumor and normal samples, which included 202 samples of normal tissue and 307 samples of tumor tissue (S1 Table).

### Datasets processing and meta-analysis

The preprocessing steps were performed on each platform independently. Affymetrix datasets were preprocessed and normalized with the Robust Multi-Array Average (RMA) approach [9] using Expression Console (Affymetrix, Santa Clara, CA, USA). The expression values of Agilent microarray data were normalized using the Loess algorithm. The probe set IDs were mapped to gene symbols according to the probe annotation files. Next, expression values of the same gene symbols were collapsed based on the mean value of each gene in each database. In addition, genes with low expression levels and low variation in expression values were removed. After processing, to identify DEGs, meta-analysis was conducted using the Rank Prod method in the MetaDE R package [10]. Genes with a False Discovery Rate (FDR) of less than 0.05 were considered significant DEGs.

### Gene enrichment analysis

GO enrichment and KEGG pathway analyses were performed for DEGs using the g:Profiler database (https://biit.cs.ut.ee/gprofiler/gost) with an adjusted *P-value* significance level of ≤ 0.05.

### Protein-protein interaction network analysis

To investigate the interactions among the DEGs, a protein-protein interaction (PPI) network was constructed by the STRING database (http://string-db.org) with a minimum required interaction score > 0.4. The PPI network was visualized using Cytoscape (version 3.7.1) software.

### Identification of transcription factors (TFs), protein kinases (PKs) and miRNAs

Human Transcription Factors database (http://humantfs.ccbr.utoronto.ca/allTFs.php) was used to identify TFs. The identification of PKs is considered a key step towards tumor progression [11]. The GSEA database (https://www.gsea-msigdb.org) was employed to detect potential PKs among DEGs. In addition, identification of potential miRNAs that might be related to the DEGs was carried out using the miRTarBase database (http://mirtarbase.cuhk.edu.cn/php/search.php#advanced).

### *Cis*-elements analysis

CREs are one of the most important factors in regulating gene expression in various tissues and diseases. The 1500 bp upstream flanking regions of DEGs were extracted from Ensemble (https://www.ensembl.org/index.html). The MEME online tool (http://meme-suite.org/tools/meme) was used to discover conserved motifs [12]. The Tomtom tool (http://meme-suite.org/tools/tomtom) was used to define known CREs based on the motif database of JASPAR CORE 2018 [13]. The GoMo tool (http://meme-suite.org/tools/gomo) was also applied to identify possible roles and GO terms for the motifs [14].

### Weighted gene co-expression network analysis

To build the DEGs co-expression network and to identify highly correlated genes, we used the WGCNA R package [15]. Firstly, the matrix of the normalized expression values of DEGs was used to calculate Pearson’s correlation coefficient between gene pairs. Next, this similarity matrix was transformed into a topological overlap measure (TOM). A hierarchical clustering tree was constructed and modules were detected with the cutreeDynamic function by cut-off a minimum module size of 30 genes. Subsequently, the network was visualized by using cytoscape software and hub genes were identified by using the cytoHubbo plug-in [16]. In addition, GO and KEGG pathway analyses of modules were conducted with DAVID under a significance threshold of FDR < 0.05 (https://david.ncifcrf.gov/).

### Survival analysis for identifying biomarker genes

To explore the potential prognostic value of hub genes, we used the Gene Expression Profiling Interactive Analysis (GEPIA) database (http://gepia.cancer-pku.cn/) for PDAC to perform an overall survival analysis using Mantel-Cox tests. Log-rank tests were used to determine statistical significance and Log-rank P < .05 was considered significant. Furthermore, the hazard ratio (HR) was calculated based on the Cox Proportional-Hazards Model.

## Results

### Identification of DEGs

The R package MetaDE was utilized to identify DEGs. DEGs were filtered by the criterion of FDR < 0.05. Consequently, a total of 1074 DEGs, including 535 up- and 539 down-regulated genes, were identified (S2 Table). GO analysis of the DEGs revealed significant enrichment in the GO terms for 109 Biological Processes (BPs), 38 Cellular Components (CCs), 22 Molecular Functions (MFs), and also 2 KEGG pathways (adjusted *P-value* < 0.01 as cut-offs) (S3 Table). BPs include regulation of catalytic activity, metabolism of proteins, cellular response to organic substances, regulation of developmental processes, regulation of cell migration, anatomical structure morphogenesis, regulation of molecular functions, regulation of cellular component movements, and regulation of cell motility. The MFs of DEGs are mainly concentrated on protein binding, enzyme regulator activity, catalytic activity, acting on a protein, protein kinase activity, and protein serine/threonine kinase activity. The top and significant enriched KEGG pathways were HIF-1 signaling pathway and focal adhesion (Table 1).

**Table 1:** KEGG pathways that are associated with differentially expressed genes (DEGs).

Pathway	Gene count	Adjusted *P-value*
HIF-1 signaling pathway	22	0.0002
Focal adhesion	30	0.0015

### Identification of the TFs, PKs, and miRNAs

**Fig 2:**
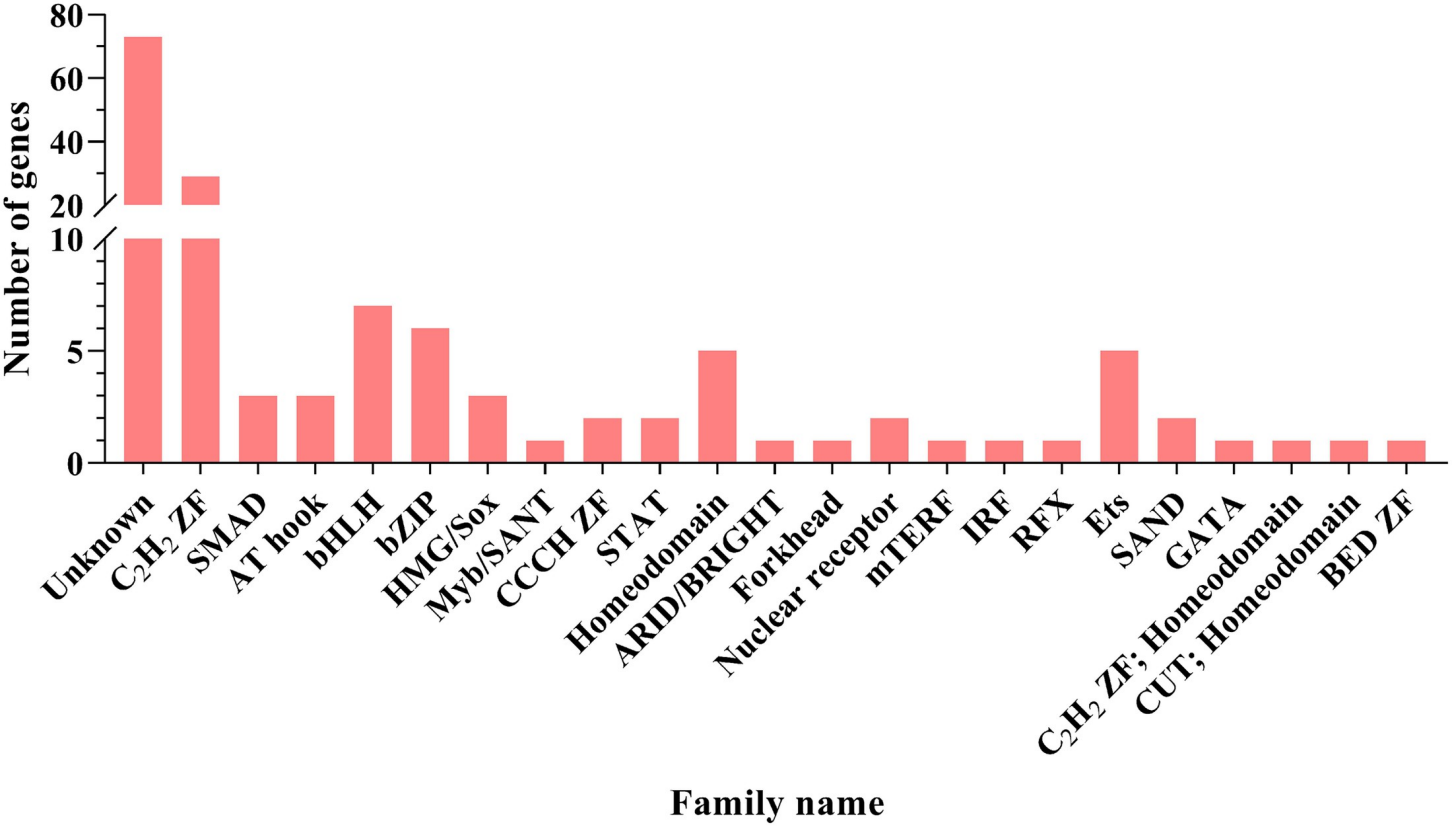
Number of differentially expressed genes (DEGs) in each TF family.

During tumorigenesis, some transcription factors cause overexpression or suppression of target genes, as well as changes in the biology of cancer cells. As a result, targeting of transcription factors is a possible strategy for cancer therapy. Among DEGs, 152 TFs belonged to 23 conserved families were identified, whereas 2 families were the largest groups. One is unknown and contains 73 genes and the other is *C2H2 ZF* with 29 genes (Fig 2). Sixty-four protein kinase gene were identified and classified into 12 families. The *CAMK* was the largest group among these families (Table 2 and S4 Table). A total of 39 and 25 PKs were up- and down-regulated, respectively.

**Table 2:** List of protein kinase families.

Family	Number of genes	Genes
Tyr	7	*ABL2*, *EGFR*, *ERBB3*, *FGFR1*, *IGF1R*, *INSR*, *DDR2*,
TKL	3	*BMPR2*, *TGFBR1*, *RIPK4*
CAMK	12	*CAMK2G*, *DAPK3*, *MKNK2*, *MYLK*, *STK11*, *TRIO*, *MKNK1*, *CASK*, *STK17B*, *SIK2*, *PRKD2*, *CAMK1D*
STE	11	*MAP3K8*, *PAK1*, *PAK3*, *STK3*, *STK4*, *STK10*, *MAP3K12*, *MAP3K13*, *MAP4K4*, *TNIK*, *STK26*
CMGC	8	*CDK1*, *CDK6*, *EFEMP1*, *MAPK6*, *MAPK9*, *HIPK2*, *NLK*, *CLK4*
AGC	4	*PRKCE*, *CDC42BPA*, *CIT*, *MAST4*
CK1	1	*CSNK1G3*
Atypical: PI3/PI4-kinase family	2	*ATR*, *PIK3CA*
Atypical: PDK/BCKDK protein kinase family	2	*PDK1*, *PDK4*
Atypical: Alpha-type protein kinase family	1	*ALPK1*
Atypical: RIO-type Ser/Thr protein kinase family	1	*RIOK3*
Other	12	*PLK3*, *ERN1*, *EIF2AK2*, *RNASEL*, *PPM1D*, *TGFBR3*, *HTATIP2*, *EIF2AK3*, *ERN2*, *MMD*, *WNK1*, *PEAK1*

We investigated potential miRNAs that may be related to DEGs using the mirtarbase database. A total of 901 miRNAs, which belonged to 195 families, were found. Among the detected miRNAs, the hsa-miR-200 family comprised the highest frequency with 59 members (Table 3 and S5 Table).

**Table 3:** Twenty-five top mirRNAs that target differentially expressed genes (DEGs).

miRNA Family	Count of Genes
hsa-miR-200	59
hsa-let-7	36
hsa-miR-29	30
hsa-miR-34	26
hsa-miR-125	24
hsa-miR-133, hsa-miR-7	18
hsa-miR-26, hsa-miR-16, hsa-miR-146, hsa-miR-27	17
hsa-miR-155	15
hsa-miR-124, hsa-miR-181	14
hsa-miR-145, hsa-miR-15, hsa-miR-223	13
hsa-miR-203, hsa-miR-30	12
hsa-miR-21	11
hsa-miR-107, hsa-miR-137, hsa-miR-199	10
hsa-miR-375, hsa-miR-23	9

### *Cis*-elements analysis

Conserved motifs and consensus CREs were detected by analyzing the 1500 bp upstream flanking regions. Eleven significant motifs were detected by the MEME database (Table 4) and further motif enrichment was performed by using the GOMO tool (S6 Table). Gene ontology indicated that these motifs participated in sensory perception of smell, DNA damage checkpoint, regulation of organ growth, translational elongation, and RNA processing. These motifs were also involved in molecular functions such as olfactory receptor activity, structural constituent of ribosome, transcription factor activity, histone binding, proton-transporting ATPase activity, and rotational mechanism (Table 4).

**Table 4:** The conserved motifs found in promoter of differentially expressed genes (DEGs).

Motif	E-value	Width	Best match in JASPAR	Significant GO term identified by GOMO
Motif 1	2.7e-346	29	MA1281.1	MF olfactory receptor activity
				BP sensory perception of smell
				BP DNA damage checkpoint
				BP regulation of organ growth
				MF proton-transporting ATPase activity, rotational mechanism
Motif 2	1.70E-295	31	MA0631.1	MF olfactory receptor activity
				BP sensory perception of smell
Motif 3	8.00E-280	21	MA0234.1	CC mitochondrion
				BP RNA metabolic process
				MF structural constituent of ribosome
				BP translational elongation
				CC ribosome
Motif 4	4.2e-394	36	MA0814.1	MF histone binding
				BP translational elongation
				CC cytosolic ribosome
				MF structural constituent of ribosome
				BP sensory perception of smell
Motif 5	3.30E-220	33	MA1242.1	MF transcription factor activity
				BP regulation of transcription from RNA
				polymerase II promoter
				BP RNA splicing
				CC intracellular membrane-bounded organelle
Motif 6	6.40E-211	21	MA1274.1	MF olfactory receptor activity
				BP sensory perception of smell
				BP DNA damage checkpoint
				BP regulation of organ growth
				CC mitochondrion
Motif 7	5.70E-157	15	MA1274.1	MF olfactory receptor activity
				BP sensory perception of smell
				BP RNA processing
				BP negative regulation of alpha-beta T cell differentiation
				BP regulation of organ growth
Motif 8	6.00E-153	29	MA0373.1	MF olfactory receptor activity
				BP sensory perception of smell
				CC cytosolic ribosome
				BP chemotaxis
				BP translational elongation
Motif 9	1.90E-158	29	MA0212.1	MF structural constituent of ribosome
				CC mitochondrial inner membrane
				BP translational elongation
				BP nuclear mRNA splicing, via spliceosome
				CC intracellular organelle lumen
Motif 10	6.70E-184	27	MA0299.1	BP glucose catabolic process
				MF olfactory receptor activity
				BP sensory perception of smell
Motif 11	1.00E-177	40	MA0505.1	BP spliceosome assembly
				BP DNA damage checkpoint
				CC cytosolic ribosome

### WGCNA and identification of modules

**Fig 3:**
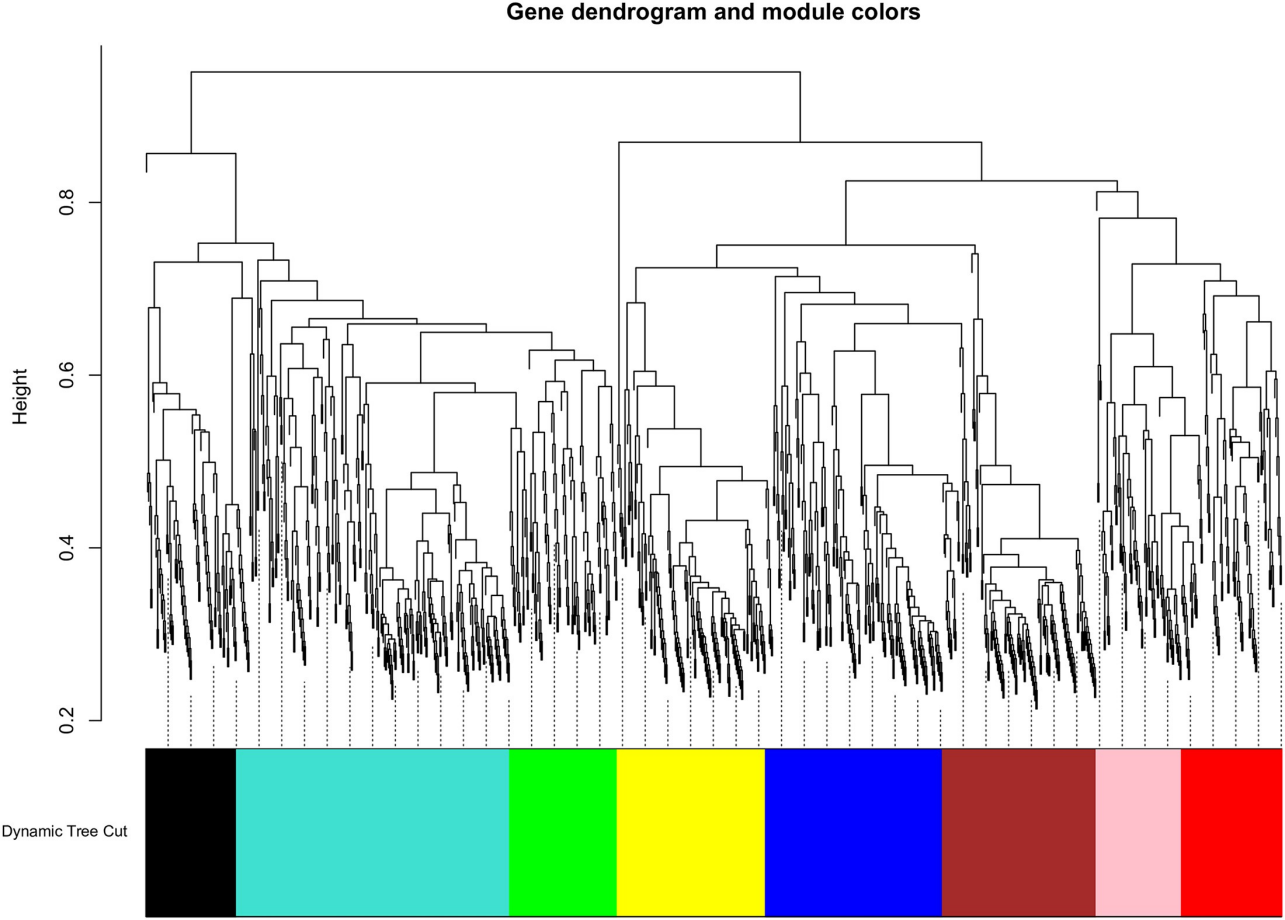
Clustering dendrograms and modules identified by weighted gene co-expression network analysis (WGCNA). The dendrogram indicates the gene clustering based on the TOM dissimilarity measure and each line indicated a gene. The colored column below the dendrogram indicates the modules conducted by the static tree cutting method at module size of 30 resulted in 8 color-coded modules.

We used the WGCNA approach to examine gene co-expression patterns in pancreatic cancer mRNA expression profiles. Initially, a similarity matrix was calculated based on Pearson correlation between each DEG pair, which was converted to a proximity matrix using a power function (β). Then, the topological overlap matrix (TOM) was calculated for hierarchical clustering analysis. Finally, a dynamic tree cutting algorithm was implemented to identify gene expression modules. The parameters used in this study were β power of 12 and a minimum module size of 30. Eventually, the DEGs were based on the dynamic tree cutting algorithm grouped into eight modules, which were labelled by different colors (turquoise, brown, blue, black, yellow, red, green, and pink). The modules ranged in size from 80 (pink module) to 256 (turquoise module) genes (Fig 3 and S7 and S8 Tables).

**Fig 4:**
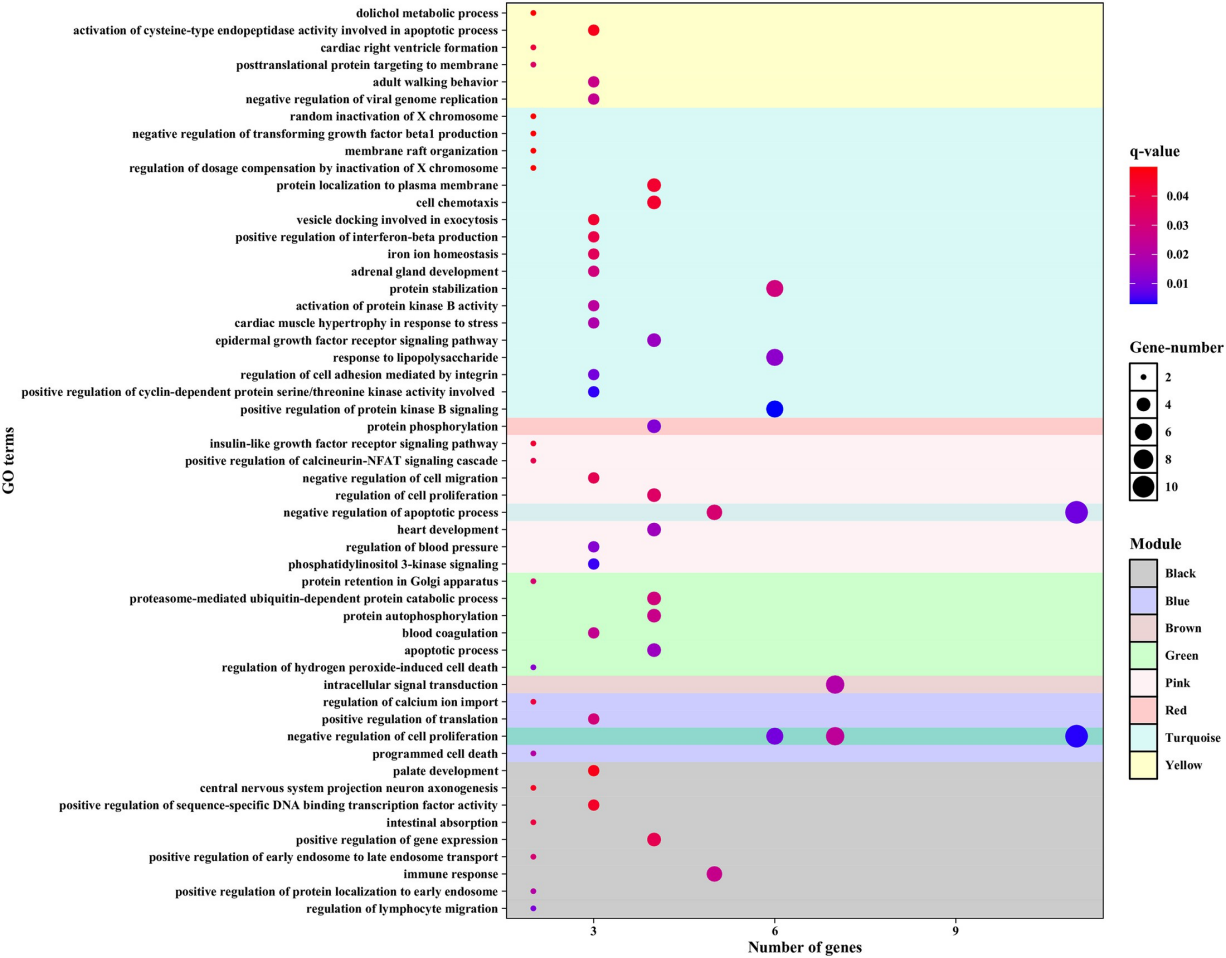
Fifty-three significant biological processes were identified in eight modules with P-value<0.05.

**Fig 5:**
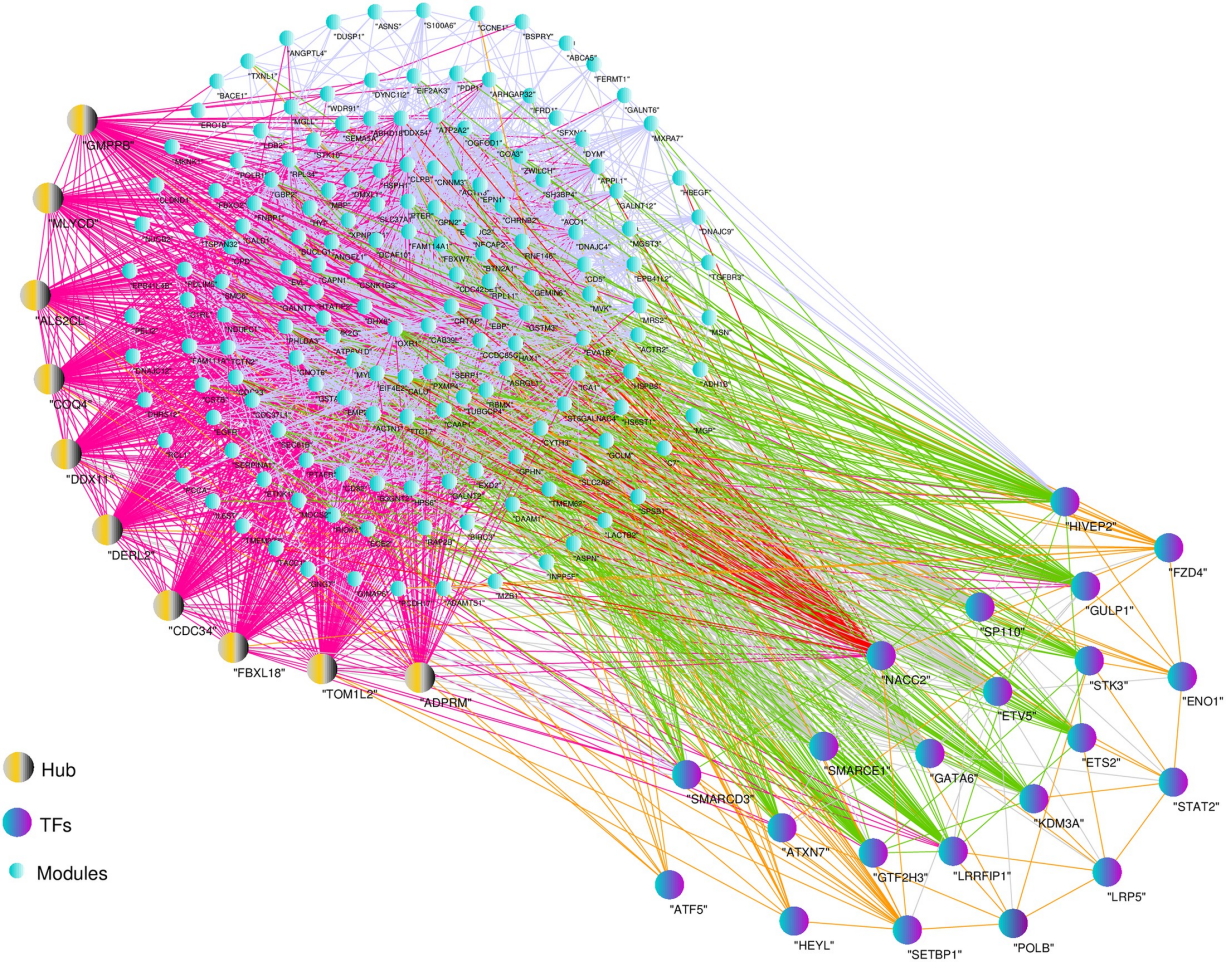
Correlation of TFs with turquoise module.

To understand the biological functions associated with modules, the enrichment analysis for the BP category was conducted by using DAVID (FDR< 0.05). The results revealed that modules were more involved in the Ras signaling pathway, p53 signaling pathway, MAPK signaling pathway, protein processing in endoplasmic reticulum, proteoglycans in cancer, focal adhesion, Rap1 signaling pathway, PI3K-Akt signaling pathway, HIF-1 signaling pathway, FoxO signaling pathway, ErbB signaling pathway, and insulin signaling pathway (Fig 4 and S9 Table). Among the modules, the highest number of TFs belonged to the turquoise module, with 22 TFs, which can indicate the regulatory role of this module (Fig 5). Moreover, a total of 11 PKs were identified in the turquoise module (S10 Table).

### Hub genes analysis in modules

To identify genes with central roles in the network, Cytoscape’s CytoHubbo plugin was used to select genes with high connectivity within each module, known as hub genes. In the CytoHubbo plugin, the top 10 nodes calculated by the maximal clique centrality (MCC) algorithm were shown as hub genes in each module. Finally, from eight modules, 80 hub genes were selected (S11 Table). The hub genes were found to be significantly enriched in 12 GO terms and 4 pathways (S12 Table). These hub genes were enriched in negative regulation of adiponectin secretion, and cell division. The turquoise module yielded the highest number of hub TFs. The *EMP1*, *ELP5*, *ABCC3*, *PIGN*, *LTBP3*, *PANX1*, *DERL2* and *RPS5* genes in the modules indicated top-ranking in each module (Table 5).

**Table 5:** Top first hub for each module.

Hub gene	Module
*EMP1*	Black
*ELP5*	Blue
*ABCC3*	Brown
*PIGN*	Green
*LTBP3*	Pink
*PANX1*	Red
*DERL2*	Turquoise
*RPS5*	Yellow

### Survival analysis of hub genes

**Fig 6:**
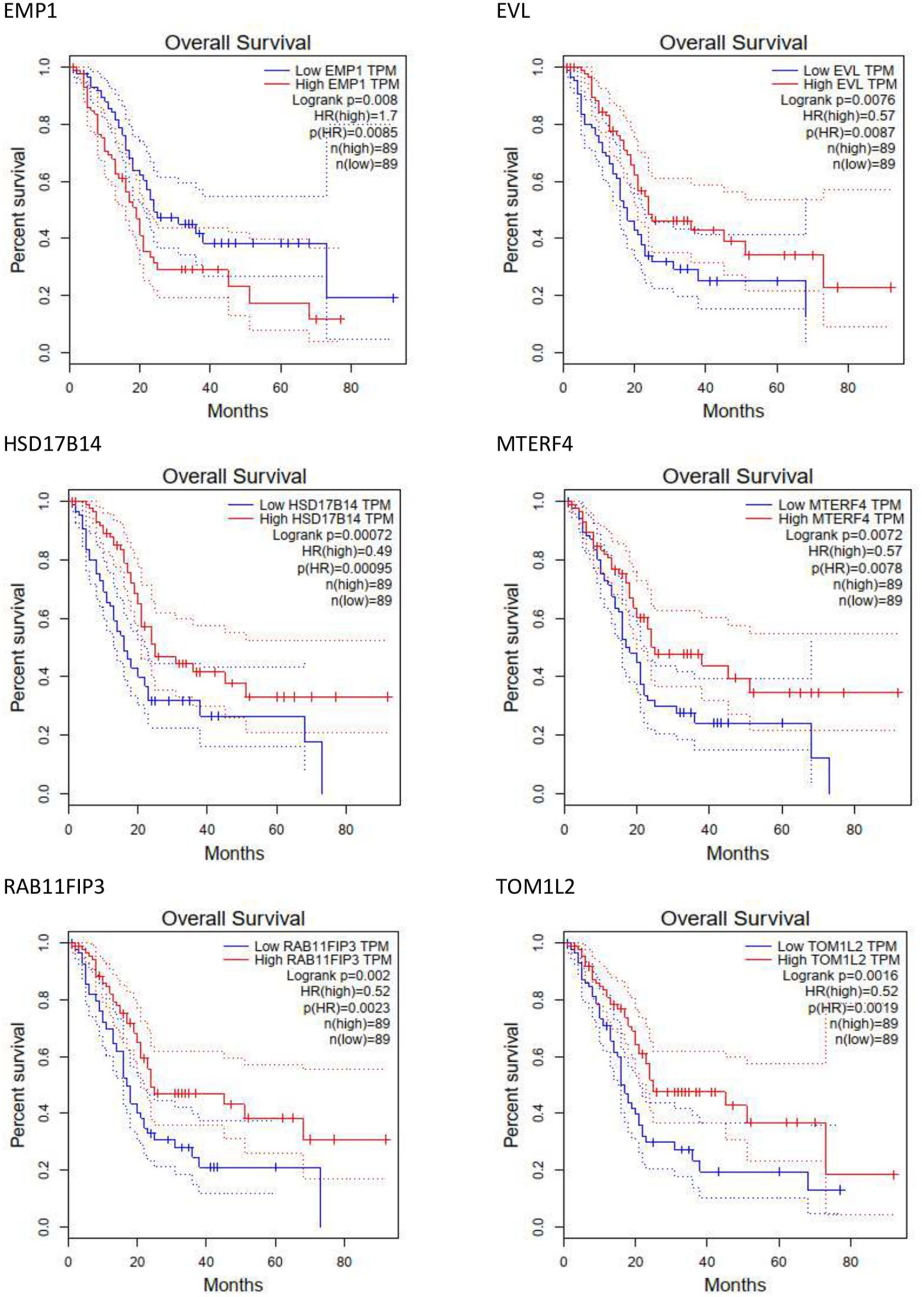
Survival analysis of six signature genes based on GEPIA data.

Survival analyses to analyze the correlation between hub gene expression and the prognosis of PDAC have been performed by using the GEPIA tool. *EMP1*(log-rank p = 0.008), *EVL* (log-rank p = 0.0076), *HSD17B14* (log-rank p = 0.00072), *MTERF4* (log-rank p = 0.0072), *RAB11FIP3* (log-rank p = 0.002), and *TOM1L2* (log-rank p = 0.0016) were the only genes with significant overall survival (log-rank P < .05) (Fig 6).

## Discussion

Pancreatic ductal adenocarcinoma is a gastrointestinal malignant tumor that is diagnosed at an advanced stage due to a lack of effective screening tools and biomarkers. As a result, PDCA patients have a low survival rate. Therefore, the identification of reliable biomarkers associated with prognosis and treatment in PDCA is critical. There is a large amount of transcriptome data available, which allows researchers to identify biomarkers and metabolic pathways involved in various cancers [17]. In this regard, we collected 509 samples of microarray data from different datasets. Finally, 1074 DEGs were screened out by meta-analysis that had different expression levels in tumor tissues. The pathway enrichment analysis of DEGs showed that some enriched terms were related to the HIF-1 signaling pathway (KEGG:04066) and focal adhesion (KEGG:04510). HIF-1 has been reported to be involved in human cancers such as ovarian, prostate, and breast cancers. Deng et al., found that HIF-1 signaling increases in hepatocellular carcinoma compared to normal tissue and also plays a major role in cancer prognosis [18]. HIF-1α is a major factor involved in the regulation of cellular responses in prostate cancer; it is activated and decreased by hypoxia and targets the HIF pathway [19]. On the other hand, cancer cells consume oxygen, disrupt oxygen balance, and cause hypoxia, while cell growth and proliferation result in an increase in oxygen [20]. In fact, with increasing and decreasing oxygen levels, conditions are created for tumor growth and the survival of cancer cells increases [21]. However, oxygen in eukaryotic cells is essential for aerobic metabolism and ATP production. Therefore, it is important to maintain oxygen homeostasis. Focal adhesions play important roles in biological processes such as cell motility, cell proliferation, cell differentiation, regulation of gene expression, and cell survival [22]. They are communicators and adhesion between cells and the extracellular matrix (ECM). Focal adhesion kinase (FAK), a cytoplasmic non-receptor tyrosine kinase, is a key regulator in FAs, which leads to FA signals on cell adhesion to the ECM [23]. It has been reported that FAK is expressed in pancreatic cancer cell lines at the levels of mRNA, protein, and phosphorylated protein. It has previously been shown that FAK knockdown and FAK kinase inhibition have antitumor activity [24].

To study the regulatory mechanisms, we identified transcription factors among DEGs. The Cys2-His2 zinc finger family (C2H2-ZF) was the largest of the 152 TFs discovered. *Cys2-His2* zinc finger (C2H2-ZF) proteins are the largest class of putative human transcription factors. Najafabadi et al. have indicated that the human genome contains an extensive and largely unexplored *C2H2-ZF* regulatory network that targets various genes and pathways [25]. Munro et al. have found that somatic mutations within *Cys2His2* zinc finger genes lead to widespread transcriptional dysregulation in cancer cells [26]. In the present study, *EGR1* was identified as one of the TFs that belong to the family of C2H2-ZF. *EGR1* is involved in tumor cell proliferation, invasion and metastasis, and tumor angiogenesis. It has also been reported that δ-tocotrinol in pancreatic cancer cells stimulates the expression of *EGR1*, which causes apoptosis of pancreatic cancer cells [27].

The results of meta-analysis were further investigated for protein kinases. Protein kinases are kinase enzymes that phosphorylate a target protein to change its function. Protein kinases have been shown in studies to be important cancer regulators [28]. In this study, we identified several protein kinase genes that associate with pancreatic cancer. Sixty-four protein kinases from 12 families were identified. The largest families included CAMK, TyR, STE and CMGC. STKs, which are members of the STE family, are enzymes that modulate protein activity by phosphorylating the serine and threonine amino acids [29]. This family is involved in signal transduction pathways, controlling metabolism, cell division, and angiogenesis [30]. They have also been shown to be involved in various types of cancer. For example, STK 4 is involved in pancreatic [31] and colorectal cancers [32, 33]. In addition, epidermal growth factor–containing fibulin-like extracellular matrix protein 1 (*EFEMP1*) as a member of the CMGCs family was detected.

*EFEMP1* is involved in anti-angiogenic via suppression of endothelial cell sprouting [34]. Changes in *EFEMP1* expression have previously been linked to cancers such as lung, liver, breast, prostate, nasopharyngeal, and pancreatic adenocarcinoma [35–41].

Considering the importance of miRNAs in cancer, we also identified miRNAs associated with DEGs. A total of 901 miRNA-related genes belong to 195 families, which may have important roles in pancreatic cancer. miRNAs can affect the expression profiles of genes such as oncogenes and tumor suppressor genes, as well as cause the formation and progression of cancer [42]. Hsa-miR-200 was the largest family, with 59 members. Peng et al. reported that the miR-200 family is involved in the onset and metastasis of cancer [43]. According to Barshack et al., the hsa-miR-200 family expression has been significantly increased in liver malignancies, which can be used in tumor diagnosis [44]. Moreover, Yu et al. suggested miR-200c as a new marker for the prognosis of pancreatic cancer [45].

We also performed a promoter analysis to identify regulatory elements upstream of the DEGs. Eleven motifs with significant scores were discovered. A large number of olfactory receptor activity–associated motifs were detected in the DEG upstream promoter sequences. Olfactory receptors (ORs) are a large group of G protein-coupled receptors in the olfactory epithelium [46]. ORs are expressed ectopically in many tissues, and some evidence points to the role of ORs in several diseases, including cancer [47]. They are also involved in various physiological processes such as cell migration, proliferation, and secretion [48]. They have also been mentioned in several studies as biomarkers for various cancer tissues such as breast cancer [47, 48], bladder cancer [46], and small intestine neuroendocrine carcinomas [49].

WGCNA was performed using the DEGs obtained from meta-analysis to identify the co-expressed and hub genes, and a total of 8 modules were discovered. From each module, ten hub genes were extracted. Based on GO enrichment analysis, the hub genes act as regulators of cell proliferation as well as regulators of apoptosis and cell death. Eleven of the hub genes (*EMP1*, *EVL*, *ELP5*, *DEF8*, *MTERF4*, *GUlP1*, *CAPN1*, *IGF1R*, *HSD17B14*, *TOM1L2* and *RAB11FIP3*) were related to apoptosis, suggesting that the genes may be a potential target in PDCA. The survival analysis of these 11 genes revealed that *EMP1*, *EVL*, *HSD17B14*, *MTERF4*, *RAB11FIP3*, and *TOM1L2* expression are closely related to the prognosis of patients with PDAC. It was found that *EMP1* (epithelial membrane protein 1) encodes a protein located in the cell membrane. This gene is involved in biological processes such as cell death, epidermal development, and bubble assembly [50]. In a study by Liu et al., *EMP1* was reported to be expressed in a large number of tumors and was shown to be a cellular linkage on cell membranes and to play an important role in proliferation, invasion, metastasis of tumor cells, and mesenchymal epithelial transmission. Furthermore, *EMP1* has been shown in several studies to be a reliable biomarker in cancers such as gastric [51], colorectal [52] ovarian [53], bladder urothelial carcinoma [54] and non-small lung carcinoma [55]. *EVL* is a member of the Ena / VASP family of proteins involved in the regulation of the actin cytoskeleton. Changes in cytoskeletal composition either stimulate or suppress tumor cell invasion and migration. Mouneimne et al. showed that *EVL* decreased the migration and invasion of tumor cells. Decreased *EVL* expression in human tumor cells is also associated with high invasive activity, increased protrusion, decreased contraction and adhesion [56]. This gene is also involved in cervical cancer [57]. The *HSD17B14* gene encodes 17β- Hydroxysteroid dehydrogenase. Sivik et al. discovered that *HSD17B14* is a predictor marker for the tamoxifen response in breast cancer [58]. The *MTERF* family includes *MTERF1*, *MTERF2*, *MTERF3*, and *MTERF4* that have roles in the pathogenesis of various cancer types. In a study by Sun et al., it was indicated that high mRNA expression levels of the *MTERF* family lead to an improved overall survival (OS) rate in patients with lung adenocarcinoma. Furthermore, in their study, they identified *MTERFs* as primary biomarkers for predicting non-small cell lung cancer [59]. *RAB11FIP3* is an interaction of RAB11GTPase with the FIP3 protein. RAB11 GTPase is a major regulator of vesicle trafficking and belongs to a family of proteins that are susceptible to changes in human cancers [60]. Tong et al. showed that *RAB11FIP3* is involved in the endocytosis recycling in breast cancer and promotes EGFR transmission [61]. The next gene in the list, Tom1l2, belongs to the Tom1 family that may be involved in the immune response and suppression of tumors [62].

## Conclusions

In conclusion, in this study, several bioinformatics methods were used to identify novel biomarkers for pancreatic cancer. 1074 DEGs were screened and TFs, PKs, miRNAs, and regulatory elements were identified by analysis. Following that, among the DEGs, 11 important hub genes were found that were associated with many pathways of tumor progression. Among them, *EMP1* and *RAB11FIP3* were identified as new biomarkers for the treatment and prognosis of patients with PDAC. A comprehensive study is required in future research to confirm the prognostic and diagnostic value of the identified biomarkers. Therefore, they may be promising prognostic indicators for patients with PDAC.

## Supporting information

S1 Fig (PDF)

S1 Table (XLSX)

S2 Table (XLSX)

S3 Table (XLSX)

S4 Table (XLSX)

S5 Table (XLSX)

S6 Table (XLSX)

S7 Table (XLSX)

S8 Table (XLSX)

S9 Table (XLSX)

S10 Table (XLSX)

S11 Table (XLSX)

S12 Table (XLSX)

## References

[1] Pothuraju R, Rachagani S, Junker WM, Chaudhary S, Saraswathi V, Kaur S, et al. Pancreatic cancer associated with obesity and diabetes: an alternative approach for its targeting. J Exp Clin Cancer Res. 2018;37(1):319. Epub 2018/12/21. doi: 10.1186/s13046-018-0963-4 . 30567565 PMC6299603

[2] Sung H, Ferlay J, Siegel RL, Laversanne M, Soerjomataram I, Jemal A, et al. Global Cancer Statistics 2020: GLOBOCAN Estimates of Incidence and Mortality Worldwide for 36 Cancers in 185 Countries. CA Cancer J Clin. 2021;71(3):209–49. Epub 2021/02/05. doi: 10.3322/caac.21660 . 33538338

[3] Wang W, Xing H, Huang C, Pan H, Li D. Identification of pancreatic cancer type related factors by Weighted Gene Co-Expression Network Analysis. Med Oncol. 2020;37(4):33. Epub 2020/03/23. doi: 10.1007/s12032-020-1339-0 . 32200436

[4] Irigoyen A, Jimenez-Luna C, Benavides M, Caba O, Gallego J, Ortuno FM, et al. Integrative multi-platform meta-analysis of gene expression profiles in pancreatic ductal adenocarcinoma patients for identifying novel diagnostic biomarkers. PLoS One. 2018;13(4):e0194844. Epub 2018/04/05. doi: 10.1371/journal.pone.0194844 . 29617451 PMC5884535

[5] Goonesekere NC, Wang X, Ludwig L, Guda C. A meta analysis of pancreatic microarray datasets yields new targets as cancer genes and biomarkers. PLoS One. 2014;9(4):e93046. Epub 2014/04/18. doi: 10.1371/journal.pone.0093046 . 24740004 PMC3989178

[6] Usadel B, Obayashi T, Mutwil M, Giorgi FM, Bassel GW, Tanimoto M, et al. Co-expression tools for plant biology: opportunities for hypothesis generation and caveats. Plant Cell Environ. 2009;32(12):1633–51. Epub 2009/08/29. doi: 10.1111/j.1365-3040.2009.02040.x . 19712066

[7] Su Q, Zhu EC, Qu YL, Wang DY, Qu WW, Zhang CG, et al. Serum level of co-expressed hub miRNAs as diagnostic and prognostic biomarkers for pancreatic ductal adenocarcinoma. J Cancer. 2018;9(21):3991–9. Epub 2018/11/10. doi: 10.7150/jca.27697 . 30410604 PMC6218787

[8] Giulietti M, Occhipinti G, Principato G, Piva F. Identification of candidate miRNA biomarkers for pancreatic ductal adenocarcinoma by weighted gene co-expression network analysis. Cell Oncol (Dordr). 2017;40(2):181–92. Epub 2017/02/17. doi: 10.1007/s13402-017-0315-y . 28205147 PMC13001553

[9] McCall MN, Bolstad BM, Irizarry RA. Frozen robust multiarray analysis (fRMA). Biostatistics. 2010;11(2):242–53. Epub 2010/01/26. doi: 10.1093/biostatistics/kxp059 . 20097884 PMC2830579

[10] Phan JH, Young AN, Wang MD. Robust microarray meta-analysis identifies differentially expressed genes for clinical prediction. ScientificWorldJournal. 2012;2012:989637. Epub 2013/02/01. doi: 10.1100/2012/989637 . 23365541 PMC3539384

[11] Ghione S, Mabrouk N, Paul C, Bettaieb A, Plenchette S. Protein kinase inhibitor-based cancer therapies: considering the potential of nitric oxide (NO) to improve cancer treatment. Biochemical pharmacology. 2020;176:113855. doi: 10.1016/j.bcp.2020.113855 32061562

[12] Bailey TL, Boden M, Buske FA, Frith M, Grant CE, Clementi L, et al. MEME SUITE: tools for motif discovery and searching. Nucleic Acids Res. 2009;37(Web Server issue):W202–8. Epub 2009/05/22. doi: 10.1093/nar/gkp335 . 19458158 PMC2703892

[13] Gupta S, Stamatoyannopoulos JA, Bailey TL, Noble WS. Quantifying similarity between motifs. Genome Biol. 2007;8(2):R24. Epub 2007/02/28. doi: 10.1186/gb-2007-8-2-r24 . 17324271 PMC1852410

[14] Buske FA, Boden M, Bauer DC, Bailey TL. Assigning roles to DNA regulatory motifs using comparative genomics. Bioinformatics. 2010;26(7):860–6. Epub 2010/02/12. doi: 10.1093/bioinformatics/btq049 . 20147307 PMC2844991

[15] Langfelder P, Horvath S. WGCNA: an R package for weighted correlation network analysis. BMC Bioinformatics. 2008;9:559. Epub 2008/12/31. doi: 10.1186/1471-2105-9-559 . 19114008 PMC2631488

[16] Chin CH, Chen SH, Wu HH, Ho CW, Ko MT, Lin CY. cytoHubba: identifying hub objects and sub-networks from complex interactome. BMC Syst Biol. 2014;8 Suppl 4:S11. Epub 2014/12/19. doi: 10.1186/1752-0509-8-S4-S11 . 25521941 PMC4290687

[17] Tu J, Chen J, He M, Tong H, Liu H, Zhou B, et al. Bioinformatics analysis of molecular genetic targets and key pathways for hepatocellular carcinoma. Onco Targets Ther. 2019;12:5153–62. Epub 2019/07/16. doi: 10.2147/OTT.S198802 . 31303768 PMC6612290

[18] Deng F, Chen D, Wei X, Lu S, Luo X, He J, et al. Development and validation of a prognostic classifier based on HIF-1 signaling for hepatocellular carcinoma. AGING. 2020;12:4. doi: 10.18632/aging.102820 32084009 PMC7066907

[19] Song J, Chen W, Zhu G, Wang W, Sun F, Zhu J. Immunogenomic Profiling and Classification of Prostate Cancer Based on HIF-1 Signaling Pathway. Front Oncol. 2020;10:1374. Epub 2020/08/28. doi: 10.3389/fonc.2020.01374 . 32850440 PMC7425731

[20] Masoud GN, Li W. HIF-1alpha pathway: role, regulation and intervention for cancer therapy. Acta Pharm Sin B. 2015;5(5):378–89. Epub 2015/11/19. doi: 10.1016/j.apsb.2015.05.007 . 26579469 PMC4629436

[21] Galanis A, Pappa A, Giannakakis A, Lanitis E, Dangaj D, Sandaltzopoulos R. Reactive oxygen species and HIF-1 signalling in cancer. Cancer Lett. 2008;266(1):12–20. Epub 2008/04/02. doi: 10.1016/j.canlet.2008.02.028 . 18378391

[22] Nagano M, Hoshino D, Koshikawa N, Akizawa T, Seiki M. Turnover of focal adhesions and cancer cell migration. Int J Cell Biol. 2012;2012:310616. Epub 2012/02/10. doi: 10.1155/2012/310616 . 22319531 PMC3272802

[23] Bauer MS, Baumann F, Daday C, Redondo P, Durner E, Jobst MA, et al. Structural and mechanistic insights into mechanoactivation of focal adhesion kinase. Proc Natl Acad Sci U S A. 2019;116(14):6766–74. Epub 2019/03/17. doi: 10.1073/pnas.1820567116 . 30877242 PMC6452671

[24] Ucar DA, Dang LH, Hochwald SN. Focal adhesion kinase signaling and function in pancreatic cancer. Frontiers in Bioscience E3. 2011:750–6. doi: 10.2741/e283 21196348

[25] Najafabadi HS, Mnaimneh S, Schmitges FW, Garton M, Lam KN, Yang A, et al. C2H2 zinc finger proteins greatly expand the human regulatory lexicon. Nature biotechnology. 2015;33(5):555–62. doi: 10.1038/nbt.3128 25690854

[26] Munro D, Ghersi D, Singh M. Two critical positions in zinc finger domains are heavily mutated in three human cancer types. PLoS Comput Biol. 2018;14(6):e1006290. Epub 2018/06/29. doi: 10.1371/journal.pcbi.1006290 . 29953437 PMC6040777

[27] Wang C, Husain K, Zhang A, Centeno BA, Chen DT, Tong Z, et al. EGR-1/Bax pathway plays a role in vitamin E delta-tocotrienol-induced apoptosis in pancreatic cancer cells. J Nutr Biochem. 2015;26(8):797–807. Epub 2015/05/23. doi: 10.1016/j.jnutbio.2015.02.008 . 25997867 PMC4576995

[28] Kini SG, Garg V, Prasanna S, Rajappan R, Mubeen M. Protein Kinases as Drug Targets in Human and Animal Diseases. Current Enzyme Inhibition. 2017;13(2). doi: 10.2174/1573408013666161128144216

[29] Hanks SK, Hunter T. The eukaryotic protein kinase superfamily: kinase (catalytic) domain structure and classification 1. The FASEB Journal. 1995;9(8):576–96. doi: 10.1096/fasebj.9.8.7768349 7768349

[30] Ciccone MA, Maoz A, Casabar JK, Machida H, Mabuchi S, Matsuo K. Clinical outcome of treatment with serine-threonine kinase inhibitors in recurrent epithelial ovarian cancer: a systematic review of literature. Expert Opin Investig Drugs. 2016;25(7):781–96. Epub 2016/04/22. doi: 10.1080/13543784.2016.1181748 . 27101098 PMC7534810

[31] Mirus JE, Zhang Y, Hollingsworth MA, Solan JL, Lampe PD, Hingorani SR. Spatiotemporal proteomic analyses during pancreas cancer progression identifies serine/threonine stress kinase 4 (STK4) as a novel candidate biomarker for early stage disease. Mol Cell Proteomics. 2014;13(12):3484–96. Epub 2014/09/17. doi: 10.1074/mcp.M113.036517 . 25225358 PMC4256499

[32] Babel I, Barderas R, Diaz-Uriarte R, Martinez-Torrecuadrada JL, Sanchez-Carbayo M, Casal JI. Identification of tumor-associated autoantigens for the diagnosis of colorectal cancer in serum using high density protein microarrays. Mol Cell Proteomics. 2009;8(10):2382–95. Epub 2009/07/30. doi: 10.1074/mcp.M800596-MCP200 . 19638618 PMC2758763

[33] Zhai XH, Yu JK, Yang FQ, Zheng S. Identification of a new protein biomarker for colorectal cancer diagnosis. Mol Med Rep. 2012;6(2):444–8. Epub 2012/05/23. doi: 10.3892/mmr.2012.923 . 22614045

[34] Albig AR, Neil JR, Schiemann WP. Fibulins 3 and 5 antagonize tumor angiogenesis in vivo. Cancer Res. 2006;66(5):2621–9. Epub 2006/03/03. doi: 10.1158/0008-5472.CAN-04-4096 . 16510581

[35] Yue W, Dacic S, Sun Q, Landreneau R, Guo M, Zhou W, et al. Frequent inactivation of RAMP2, EFEMP1 and Dutt1 in lung cancer by promoter hypermethylation. Clin Cancer Res. 2007;13(15 Pt 1):4336–44. Epub 2007/08/03. doi: 10.1158/1078-0432.CCR-07-0015 . 17671114

[36] Nomoto S, Kanda M, Okamura Y, Nishikawa Y, Qiyong L, Fujii T, et al. Epidermal growth factor-containing fibulin-like extracellular matrix protein 1, EFEMP1, a novel tumor-suppressor gene detected in hepatocellular carcinoma using double combination array analysis. Ann Surg Oncol. 2010;17(3):923–32. Epub 2009/11/10. doi: 10.1245/s10434-009-0790-0 . 19898900

[37] Kim YJ, Yoon HY, Kim SK, Kim YW, Kim EJ, Kim IY, et al. EFEMP1 as a novel DNA methylation marker for prostate cancer: array-based DNA methylation and expression profiling. Clin Cancer Res. 2011;17(13):4523–30. Epub 2011/05/17. doi: 10.1158/1078-0432.CCR-10-2817 . 21571867

[38] Zhang Y, Wang R, Song H, Huang G, Yi J, Zheng Y, et al. Methylation of multiple genes as a candidate biomarker in non-small cell lung cancer. Cancer Lett. 2011;303(1):21–8. Epub 2011/01/25. doi: 10.1016/j.canlet.2010.12.011 . 21255913

[39] Sadr-Nabavi A, Ramser J, Volkmann J, Naehrig J, Wiesmann F, Betz B, et al. Decreased expression of angiogenesis antagonist EFEMP1 in sporadic breast cancer is caused by aberrant promoter methylation and points to an impact of EFEMP1 as molecular biomarker. Int J Cancer. 2009;124(7):1727–35. Epub 2008/12/31. doi: 10.1002/ijc.24108 . 19115204

[40] Hwang CF, Chien CY, Huang SC, Yin YF, Huang CC, Fang FM, et al. Fibulin-3 is associated with tumour progression and a poor prognosis in nasopharyngeal carcinomas and inhibits cell migration and invasion via suppressed AKT activity. J Pathol. 2010;222(4):367–79. Epub 2010/10/12. doi: 10.1002/path.2776 . 20927779

[41] Seeliger H, Camaj P, Ischenko I, Kleespies A, De Toni EN, Thieme SE, et al. EFEMP1 expression promotes in vivo tumor growth in human pancreatic adenocarcinoma. Mol Cancer Res. 2009;7(2):189–98. Epub 2009/02/12. doi: 10.1158/1541-7786.MCR-08-0132 . 19208748

[42] Duffy MJ, Sturgeon C, Lamerz R, Haglund C, Holubec VL, Klapdor R, et al. Tumor markers in pancreatic cancer: a European Group on Tumor Markers (EGTM) status report. Ann Oncol. 2010;21(3):441–7. Epub 2009/08/20. doi: 10.1093/annonc/mdp332 . 19690057

[43] Peng Y, Croce CM. The role of MicroRNAs in human cancer. Signal Transduct Target Ther. 2016;1:15004. Epub 2016/01/28. doi: 10.1038/sigtrans.2015.4 . 29263891 PMC5661652

[44] Barshack I, Meiri E, Rosenwald S, Lebanony D, Bronfeld M, Aviel-Ronen S, et al. Differential diagnosis of hepatocellular carcinoma from metastatic tumors in the liver using microRNA expression. Int J Biochem Cell Biol. 2010;42(8):1355–62. Epub 2010/07/14. doi: 10.1016/j.biocel.2009.02.021 . 20619223

[45] Yu J, Ohuchida K, Mizumoto K, Sato N, Kayashima T, Fujita H, et al. MicroRNA, hsa-miR-200c, is an independent prognostic factor in pancreatic cancer and its upregulation inhibits pancreatic cancer invasion but increases cell proliferation. Molecular Cancer. 2010;9(169). doi: 10.1186/1476-4598-9-169 20579395 PMC2909980

[46] Weber L, Schulz WA, Philippou S, Eckardt J, Ubrig B, Hoffmann MJ, et al. Characterization of the Olfactory Receptor OR10H1 in Human Urinary Bladder Cancer. Front Physiol. 2018;9:456. Epub 2018/06/06. doi: 10.3389/fphys.2018.00456 . 29867524 PMC5964926

[47] Asadi M, Ahmadi N, Ahmadvand S, Jafari AA, Safaei A, Erfani N, et al. Investigation of olfactory receptor family 51 subfamily j member 1 (OR51J1) gene susceptibility as a potential breast cancer-associated biomarker. PLoS One. 2021;16(2):e0246752. Epub 2021/02/11. doi: 10.1371/journal.pone.0246752 . 33566867 PMC7875425

[48] Weber L, Massberg D, Becker C, Altmuller J, Ubrig B, Bonatz G, et al. Olfactory Receptors as Biomarkers in Human Breast Carcinoma Tissues. Front Oncol. 2018;8:33. Epub 2018/03/03. doi: 10.3389/fonc.2018.00033 . 29497600 PMC5818398

[49] Cui T, Tsolakis AV, Li SC, Cunningham JL, Lind T, Oberg K, et al. Olfactory receptor 51E1 protein as a potential novel tissue biomarker for small intestine neuroendocrine carcinomas. Eur J Endocrinol. 2013;168(2):253–61. Epub 2012/11/28. doi: 10.1530/EJE-12-0814 . 23184910

[50] Stelzer G, Rosen N, Plaschkes I, Zimmerman S, Twik M, Fishilevich S, et al. The GeneCards Suite: From Gene Data Mining to Disease Genome Sequence Analyses. Curr Protoc Bioinformatics. 2016;54:1 30 1–1 3. Epub 2016/06/21. doi: 10.1002/cpbi.5 . 27322403

[51] Sun G, Zhao G, Lu Y, Wang Y, Yang C. Association of EMP1 with gastric carcinoma invasion, survival and prognosis. Int J Oncol. 2014;45(3):1091–8. Epub 2014/06/13. doi: 10.3892/ijo.2014.2488 . 24920167

[52] Sun GG, Wang YD, Cui DW, Cheng YJ, Hu WN. Epithelial membrane protein 1 negatively regulates cell growth and metastasis in colorectal carcinoma. World J Gastroenterol. 2014;20(14):4001–10. Epub 2014/04/20. doi: 10.3748/wjg.v20.i14.4001 . 24744589 PMC3983455

[53] Liu Y, Ding Y, Nie Y, Yang M. EMP1 Promotes the Proliferation and Invasion of Ovarian Cancer Cells Through Activating the MAPK Pathway. Onco Targets Ther. 2020;13:2047–55. Epub 2020/03/27. doi: 10.2147/OTT.S240028 . 32210572 PMC7071728

[54] Lin B, Zhang T, Ye X, Yang H. High expression of EMP1 predicts a poor prognosis and correlates with immune infiltrates in bladder urothelial carcinoma. Oncol Lett. 2020;20(3):2840–54. Epub 2020/08/13. doi: 10.3892/ol.2020.11841 . 32782602 PMC7400100

[55] Jain A, Tindell CA, Laux I, Hunter JB, Curran J, Galkin A, et al. Epithelial membrane protein-1 is a biomarker of gefitinib resistance. Proceedings of the National Academy of Sciences. 2005;102(33):11858–63. doi: 10.1073/pnas.0502113102 16087880 PMC1187965

[56] Mouneimne G, Hansen SD, Selfors LM, Petrak L, Hickey MM, Gallegos LL, et al. Differential remodeling of actin cytoskeleton architecture by profilin isoforms leads to distinct effects on cell migration and invasion. Cancer Cell. 2012;22(5):615–30. Epub 2012/11/17. doi: 10.1016/j.ccr.2012.09.027 . 23153535 PMC3500527

[57] Li XR, Chu HJ, Lv T, Wang L, Kong SF, Dai SZ. miR-342-3p suppresses proliferation, migration and invasion by targeting FOXM1 in human cervical cancer. FEBS Lett. 2014;588(17):3298–307. Epub 2014/07/30. doi: 10.1016/j.febslet.2014.07.020 . 25066298

[58] Sivik T, Gunnarsson C, Fornander T, Nordenskjold B, Skoog L, Stal O, et al. 17beta-Hydroxysteroid dehydrogenase type 14 is a predictive marker for tamoxifen response in oestrogen receptor positive breast cancer. PLoS One. 2012;7(7):e40568. Epub 2012/07/14. doi: 10.1371/journal.pone.0040568 . 22792371 PMC3391289

[59] Sun S, Wu C, Yang C, Chen J, Wang X, Nan Y, et al. Prognostic roles of mitochondrial transcription termination factors in non-small cell lung cancer. Oncol Lett. 2019;18(4):3453–62. Epub 2019/09/14. doi: 10.3892/ol.2019.10680 . 31516563 PMC6732965

[60] Ferro E, Bosia C, Campa CC. RAB11-Mediated Trafficking and Human Cancers: An Updated Review. Biology (Basel). 2021;10(1). Epub 2021/01/08. doi: 10.3390/biology10010026 . 33406725 PMC7823896

[61] Tong D, Liang YN, Stepanova AA, Liu Y, Li X, Wang L, et al. Increased Eps15 homology domain 1 and RAB11FIP3 expression regulate breast cancer progression via promoting epithelial growth factor receptor recycling. Tumour Biol. 2017;39(2):1010428317691010. Epub 2017/02/22. doi: 10.1177/1010428317691010 . 28215104

[62] Girirajan S, Hauck PM, Williams S, Vlangos CN, Szomju BB, Solaymani-Kohal S, et al. Tom1l2 hypomorphic mice exhibit increased incidence of infections and tumors and abnormal immunologic response. Mamm Genome. 2008;19(4):246–62. Epub 2008/03/18. doi: 10.1007/s00335-008-9100-6 . 18343975

