# Supplementary material for: A comprehensive meta-analysis of transcriptome data to identify signature genes associated with pancreatic ductal adenocarcinoma
Source: PLoS One. 2024 Feb 7;19(2):e0289561. doi: 10.1371/journal.pone.0289561 (PMC10849254; doi:10.1371/journal.pone.0289561)
Supplement: S1 Fig — (PDF) [file pone.0289561.s001.pdf]

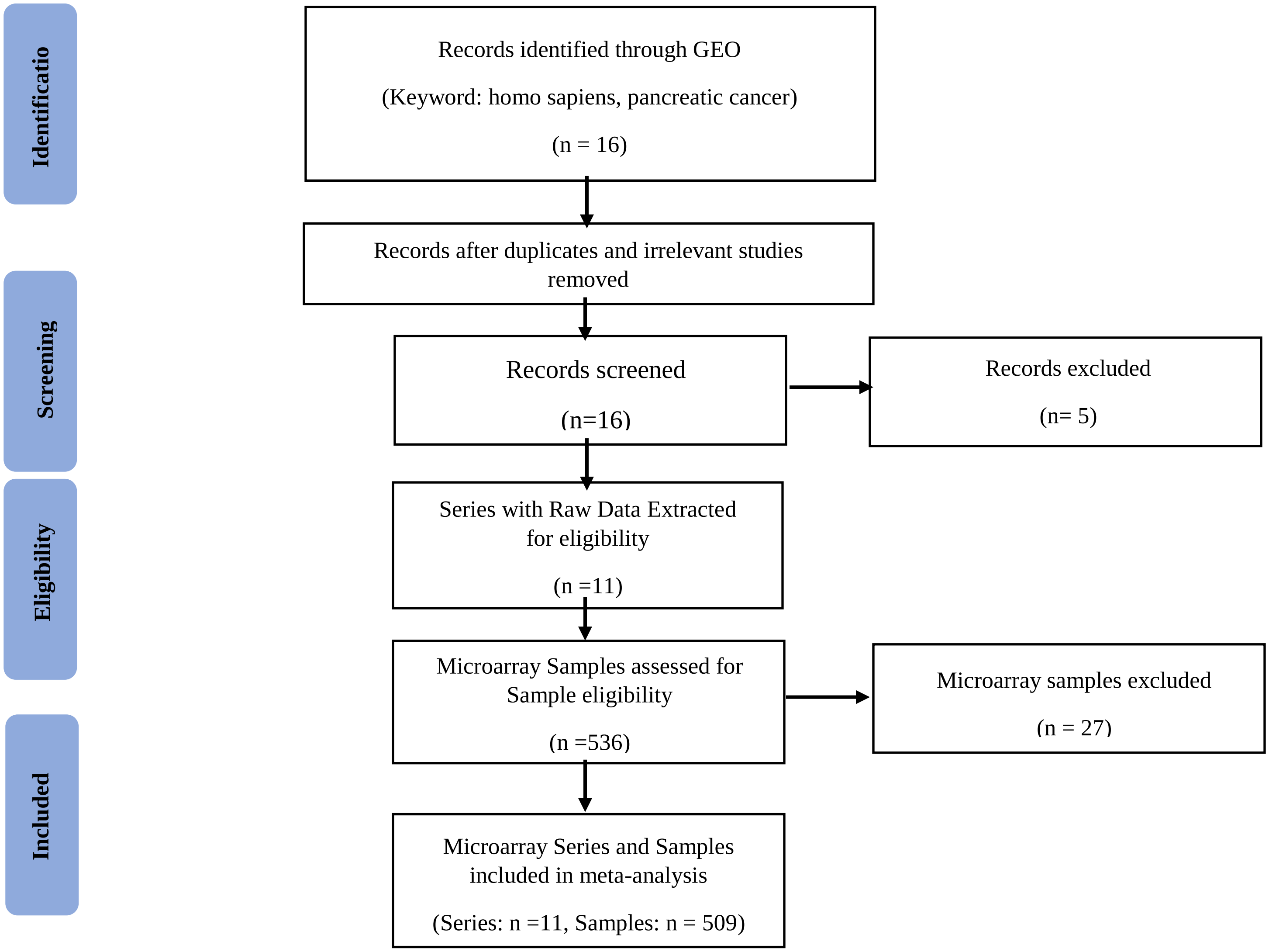

From: Page MJ, McKenzie JE, Bossuyt PM, Boutron I, Hoffmann TC, Mulrow CD, et al. The PRISMA 2020 statement: an updated guideline for reporting systematic reviews. BMJ 2021;372:n71. doi: 10.1136/bmj.n71
